# Supplementary material for: Investigating the influence of physiologically relevant hydrostatic pressure on CHO cell batch culture
Source: Sci Rep. 2021 Jan 8;11:162. doi: 10.1038/s41598-020-80576-8 (PMC7794228; doi:10.1038/s41598-020-80576-8)
Supplement: Supplementary file 1 — Supplementary Information 1. [file 41598_2020_80576_MOESM1_ESM.zip › S1/IgG/Feb14 0 30 60 90/60mmHg Feb8 2.pdf]

===

Acq. Operator : SYSTEM Seq. Line : 46
Sample Operator : SYSTEM
Acq. Instrument : HPLC-0039 Location : 12
Injection Date : 2/14/2019 5:32:23 PM Inj : 1
Inj Volume : 100.000 µl
Method : C:\Users\Public\Documents\ChemStation\1\Data\TK_CHO_IgG 2019-02-14 14-17-55
\CHO_IgG_LC.M (Sequence Method)
Last changed : 2/8/2019 3:17:23 PM by SYSTEM

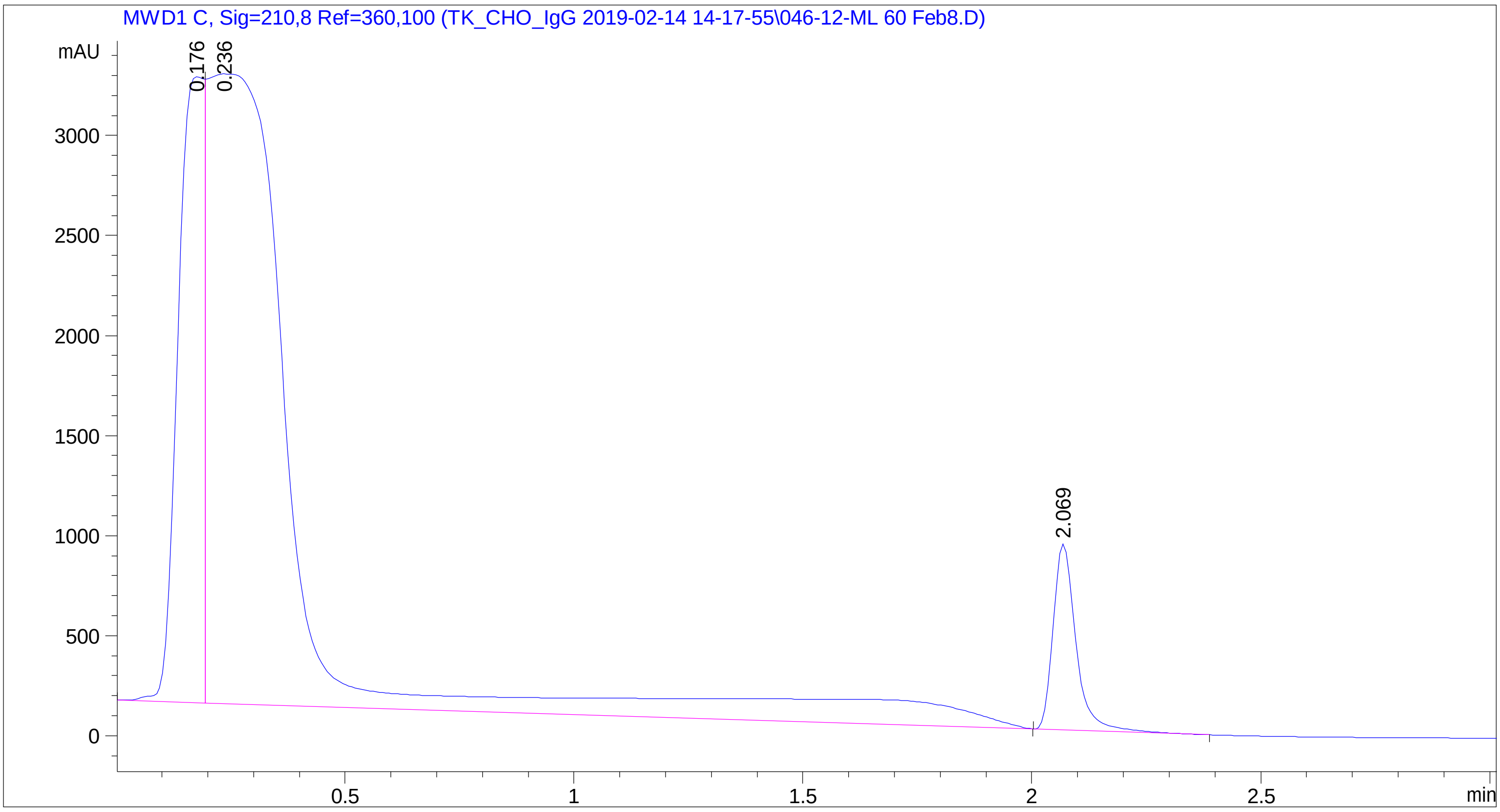

===

Area Percent Report

===

Sorted By : Signal
Multiplier : 1.0000
Dilution : 1.0000
Use Multiplier & Dilution Factor with ISTDs

Signal 1: MWD1 C, Sig=210,8 Ref=360,100

Peak #	RetTime [min]	Type	Width [min]	Area [mAU*s]	Height [mAU]	Area %
1	0.176	BV	0.0608	1.20080e4	3129.32690	21.1167
2	0.236	VB	0.1983	4.16269e4	3147.20996	73.2033
3	2.069	BBA	0.0526	3229.89160	928.03314	5.6800

Totals : 5.68647e4 7204.57001

===

*** End of Report ***
